# Intramolecular carbenoid ylide forming reactions of 2-diazo-3-keto-4-phthalimidocarboxylic esters derived from methionine and cysteine

**DOI:** 10.3762/bjoc.8.49

**Published:** 2012-03-22

**Authors:** Marc Enßle, Stefan Buck, Roland Werz, Gerhard Maas

**Affiliations:** 1 Institute of Organic Chemistry I, University of Ulm, Albert-Einstein-Allee 11, D-89081 Ulm, Germany

**Keywords:** α-aminoacids, carbonyl ylides, cycloaddition, α-diazo-β-ketoesters, sulfonium ylides

## Abstract

Methionine, *S*-benzylcysteine and *S*-allylcysteine were converted into 2-diazo-3-oxo-4-phthalimidocarboxylic esters **8a**–**c** in three steps. Upon rhodium-catalysed dediazoniation, two intramolecular carbenoid reactions competed, namely the formation of a cyclic sulfonium ylide and that of a six-ring carbonyl ylide. The *S*-methyl and *S*-benzyl ylides **12a** and **b** could be isolated, while *S*-allyl ylide **12c** underwent a [2,3]-sigmatropic rearrangement. The short-lived carbonyl ylides derived from methionine and *S*-benzylcysteine formed head-to-tail dimers by a [3 + 3]-cycloaddition and could be trapped with external dipolarophiles, while the *S*-allyl derivative **14c** yielded the pentacyclic compound **17** by an intramolecular [3 + 2]-cycloaddition reaction.

## Introduction

**Scheme 1:**
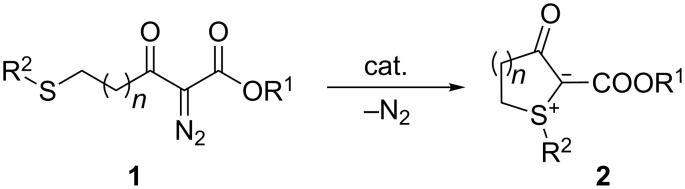
Synthesis of cyclic sulfonium ylides **2**; *n* = 0–3.

The synthetic potential of diazo compounds, in particular of α-diazoketones and α-diazoesters, is greatly widened by the ability of the derived carbene or metal-carbene intermediates to undergo inter- and intramolecular formation of N-, O-, S- and other ylides [1–2]. The benefit of these transformations, which are usually performed with rhodium- or copper-based catalysts, is given by subsequent rearrangement or addition reactions of the reactive ylides; several recent reviews [3–8] illustrate the wide range of applications. As far as sulfonium ylides are concerned, thermally induced isomerisation, that is the 1,2-shift of a substituent (Stevens rearrangement) and [2,3]-sigmatropic rearrangement of allylsulfonium ylides [9–11], and the use as C_1_ building blocks in epoxidation, aziridination and olefination reactions [12–13] are common reaction channels. The intramolecular formation of sulfonium ylides from α-diazocarbonyl compounds tethered with alkylthio or arylthio groups has been studied by the research groups of Davies [14], Moody [15], and West [16]. From α-diazo-β-ketoesters **1**, stable four- to seven-membered cyclic ylides **2** were obtained (Scheme 1); in the case of R^2^ = allyl, however, the ylides underwent a spontaneous [2,3]-sigmatropic rearrangement.

**Scheme 2:**
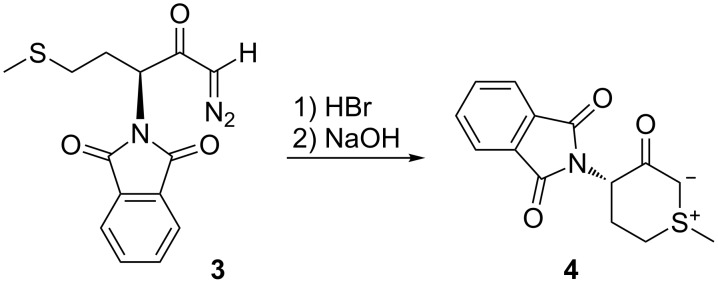
Non-carbenoid formation of sulfonium ylide **4**.

On the other hand, the conversion of methionine-derived diazoketone **3** into the cyclic sulfonium ylide **4** was not accomplished through the carbenoid route, but rather by cyclisation of a diazonium ion followed by deprotonation of the resulting sulfonium ion (Scheme 2) [17].

A diazoketone such as **3** has the structural prerequisites to undergo two types of intramolecular carbenoid ylide-forming reactions, yielding either a cyclic sulfonium ylide or a six-ring carbonyl ylide. In fact, it has been found that Rh(II)-catalyzed dediazoniation of α-diazo-β-ketoesters with γ-phthalimido [18–19] or related [20] substituents gives rise to cyclic carbonyl ylides, which were trapped by intermolecular cycloaddition reactions. In this paper, we report that the formation of sulfonium as well as carbonyl ylides are indeed competing pathways in the Rh(II)-catalyzed dediazoniation of phthaloyl-protected γ-amino-α-diazo-β-ketoesters derived from methionine, *S*-benzylcysteine, and *S*-allylcysteine.

## Results and Discussion

### Synthesis of diazoesters

**Scheme 3:**
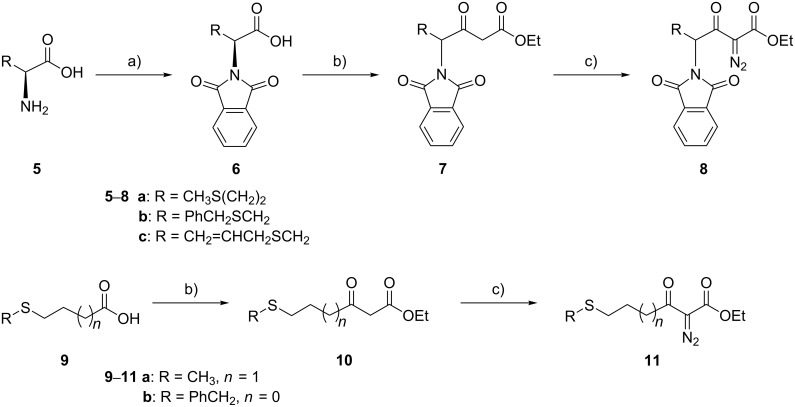
Conditions: (a) phthalic anhydride, NEt_3_ (10 mol %), toluene, reflux, 2 h; (b) 1. carbonyldiimidazole, THF, 16 h; 2. MgCl_2_, NEt_3_, HOOC–CH_2_CO_2_Et, 3.5 h; (c) *p*-tosyl azide, NEt_3_, acetonitrile, rt, 16 h (with **7a**) or imidazole-1-sulfonyl azide·HCl, NEt_3_, CH_2_Cl_2_, reflux, 16 h (with **7b,c** and **10**). Alternative synthesis of **8a**: 1. **6a**, SOCl_2_ (10 equiv), CH_2_Cl_2_, reflux, 2 h; 2. N_2_=CHCO_2_Et (2 equiv), rt, 4 days.

The 2-diazo-3-oxo-4-phthalimidocarboxylic esters **8a**–**c** were prepared from L-methionine (**5a**), *S*-benzyl-L-cysteine (**5b**) and *S*-allyl-L-cysteine (**5c**), respectively, in a three-step sequence (Scheme 3) applied by us previously for other α-aminoacids [18]. Acids **5a**–**c** were converted into their phthalimido-substituted derivatives **6** followed by conversion into β-ketoesters **7** (yields: 30% (**7a**), 60% (**7b**) and 19% (**7c**)) and diazo group transfer to give diazoesters **8** (61–99%). Phthalimido-free diazoesters **11a** and **b**, which were desired for comparative reactivity studies, were prepared analogously; it should be noted that different approaches were taken to obtain the methyl ester analogue of **11b** and other similar sulfur-containing α-diazo-β-ketoesters [14–16]. We found that diazoester **8a** can also be prepared by acylation of ethyl diazoacetate (two equivalents, one equivalent serving to trap HCl) with the acid chloride of **6a**. Although the yield was modest (28%), it was still better and gave fewer byproducts than the β-ketoester route.

The phthaloylation of **5a**–**c** was achieved with phthalic anhydride in the presence of a catalytic amount of triethylamine to lower the reaction temperature, so that the reaction could be performed in toluene under reflux. For phenylalanine, it was reported that no racemisation occurred under these conditions [21]. However, with some other α-aminoacids we have noted a small degree of racemisation [18]. In this work, we can confirm the reported specific rotation value for *N*,*N*-phthaloyl-L-methionine (**6a**), but racemisation occurred to a significant extent in the case of *S*-benzyl-*N*,*N*-phthaloyl-L-cysteine (**6b**) (Supporting Information File 1). Practically complete racemisation took place during conversion of *N*-protected aminoacids **6a–c** to the β-ketoesters **7a–c**, and therefore, diazoesters **8a–c** were also obtained in (almost) racemic form. As a consequence, diazoester **8a** was prepared subsequently from DL-methionine. On the other hand, the preparation of *N*-phthaloylated diazoester **8a** via the aminoacid chloride route (Scheme 3) seems to occur with only a little racemisation. A sample of **8a**, which was prepared from a slightly racemised sample of **6a** (HPLC: 90:10 enantiomeric ratio of the derived methyl ester, which was prepared from **6a** and dry methanol in the presence of excess chlorotrimethylsilane) on the latter route, had an enantiomeric ratio of 83:17.

### Rhodium-catalysed ylide-forming reactions

**Scheme 4:**
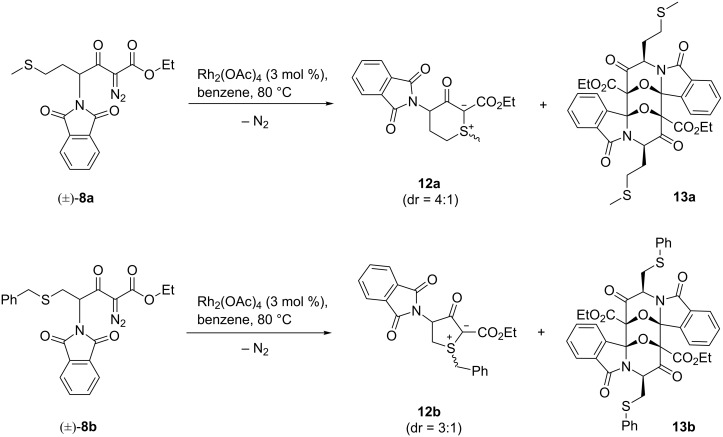
Rh(II)-catalysed carbenoid reactions of diazoesters **8a**,**b**.

A sample of racemic diazoester **8a**, prepared from DL-methionine, was exposed to a catalytic amount of dirhodium tetraacetate in boiling benzene. Two products were isolated and identified, the six-membered cyclic sulfonium ylide **12a** (13% yield) and the carbonyl ylide dimer **13a** (43%), (Scheme 4). In an analogous manner, the five-membered cyclic sulfonium ylide **12b** and polyheterocycle **13b** were formed from diazoester **8b** in yields of 37 and 25%, respectively (yields determined by ^1^H NMR integration on the crude product mixture). In contrast to these successful transformations, we found that α-diazoketone **3** (Scheme 2) was smoothly decomposed by a catalytic amount of Rh_2_(OAc)_4_ or Cu(I) triflate (e.g., 1 mol % of Rh_2_(OAc)_4_, CH_2_Cl_2_, 40 °C, 17 h), but a complex mixture of unidentified products was formed.

**Figure 1:**
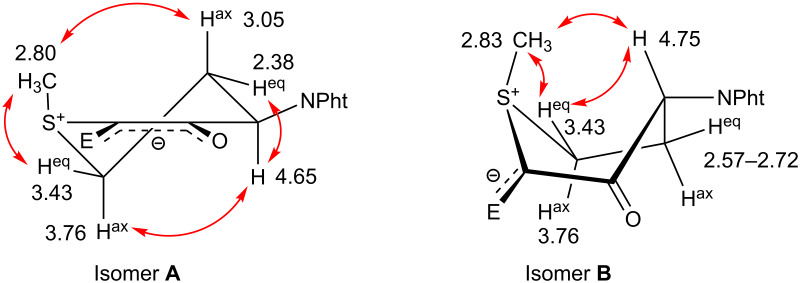
Proposed relative configurations of the diastereomeric cyclic sulfonium ylides **12aA** and **12aB**. ^1^H NMR shifts δ_H_ (ppm) and NOE relationships are shown (E = CO_2_Et).

The cyclic sulfonium ylides **12a**,**b** were both obtained as a mixture of two diastereomers. For the six-ring ylide **12a** (isomer ratio **A**:**B** = 4:1) a stereochemical assignment was made based on ^1^H NMR studies (Figure 1 and Table 1). We propose that the major isomer **12aA** assumes a cyclohexene-like half-chair conformation, and the minor isomer **12aB**, which has the opposite configuration at the phthalimido-substituted ring position, exists in a twisted-boat form. In both cases, the *S*-methyl group occupies the (pseudo-)axial position and the sterically demanding phthalimido group is in the (pseudo-)equatorial position. These conformations fit the observed nuclear Overhauser effects as well as the ^3^*J* coupling constants. Crystal structure determinations of an acyclic [22] and a five-membered cyclic [23] sulfonium ylide containing the R_2_S^+^–C^−^(COOR or COCH_3_)–C(=O)R moiety have shown that the sulfur atom is pyramidalised and the negative charge is delocalised over the π-system of the planar oxoenolate unit. This geometry agrees with the proposed conformation of **12aA**. In the twisted-boat structure of **12B**, the negative charge may be stabilised mainly by the ester group and less so by the adjacent keto group. The axial position of the methyl group at the pyramidalised sulfur atom is remarkable. In this geometry, however, p(C_ylide_)–σ^*^(S–C) conjugation of the unshared pair of electrons at the ylidic carbon atom would be feasible. While this interaction is a key stabilisation factor for the parent sulfonium ylides (e.g., H_2_S–CH_2_ and Me_2_S–CH_2_) according to theoretical calculations [24], its energetic contribution in the case of sulfonium ylides stabilised by acceptor substituents is likely to be small.

**Table 1:** ^1^H NMR data for isomers **A** (major) and **B** (minor) of cyclic sulfonium ylide **12a** (DMSO-*d*_6_, 400.13 MHz).

Assignment	Isomer A δ (ppm), multiplicity, *J* (Hz)^a^	Isomer B δ (ppm), multiplicity, *J* (Hz)^a^

OCH_2_CH_3_	1.13, t, *J* = 7.1	1.14, t, *J* = 7.1
SCH_3_	2.80, s	2.83, s
SCH^eq b^	3.41–3.46, m, ^2^*J* = 14.1, ^3^*J* = 2.8, 2.8	3.41–3.46, ddd, ^2^*J* = 13.3, ^3^*J* = 9.7, 4.1
SCH^ax b^	3.77–3.83, m, ^2^*J* ≈ ^3^*J* ≈ 14.1, ^3^*J* = 2.7	3.77–3.83, ddd, ^2^*J* = 13.3, ^3^*J* = 7.3, 4.3
SCH_2_CH^eq^	2.35–2.41 dddd, ^2^*J* = 14.1, ^3^*J* = 5.7, 2.8, 2.8	2.57–2.72, m
SCH_2_CH^ax^	3.05, dddd, ^2^*J* ≈ ^3^*J* ≈ 14.1, ^3^*J* = 12.7, ^3^*J* = 2.9	2.57–2.72, m
NCHCO	4.65, dd, ^3^*J* = 12.7, 5.7	4.75, dd, ^3^*J* = 9.1, 5.6
OCH_2_	3.94–4.07, m (both isomers)	
CH_aryl_	7.80–7.96, broadened “s” (both isomers)	

^a^Coupling constants are given without sign. ^b^The multiplet signals of SCH^eq^ and SCH^ax^ of the two isomers coincided completely. A magnetisation transfer experiment (TOCSY) allowed visualisation of the individual signal patterns.

**Scheme 5:**
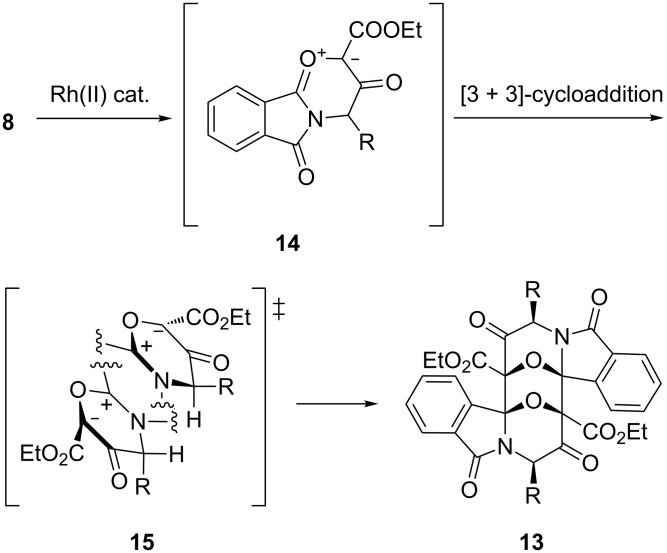
*Endo* transition state for [3 + 3]-dimerisation of carbonyl ylide **14**.

The oxazapolycycles **13a** and **b** are head-to-tail dimers of carbonyl ylides, resulting from a [3 + 3]-cycloaddition. Their constitution and relative stereochemistry was assigned by NMR comparison with the corresponding alanine-derived ring system, for which a structural proof was furnished by X-ray crystal-structure determination [18]. Due to the symmetry of the dimers, the NMR spectra display signals for only half of the total number of carbon and hydrogen nuclei. Characteristics are the signals for the NCH proton (**13a**: δ = 4.60 ppm; **13b**: δ = 4.67 ppm) and the two bridgehead carbons of the epoxy bridge (**13a**: δ = 86.60 and 94.13 ppm; **13b**: δ = 86.75 and 94.04 ppm). While a CI mass spectrum of **13b** showed a weak molecular-ion peak for the dimer (the base peak representing the monomer unit), in the case of **13a**, even under these mild conditions of chemical ionisation, the spectrum showed no peak at *m*/*z* greater than that for the monomer, which was again the base peak. The relative stereochemistry shown in Scheme 4, with the two epoxy bridges in *syn* relationship and the mercaptoalkyl substituent in the (equatorial) *exo* position, is explained by an *endo* transition state **15** for the [3 + 3]-cycloaddition, in which two cyclic carbonyl ylide molecules **14** approach each other with their sterically less-occupied faces (Scheme 5). Our findings on carbonyl ylide dimers reported here and in a preceding paper [18] tie in with earlier reports on the [3 + 3]-cyclodimerisation of carbonyl ylides of the 2-benzopyrylium-4-olate type [25–26].

**Scheme 6:**
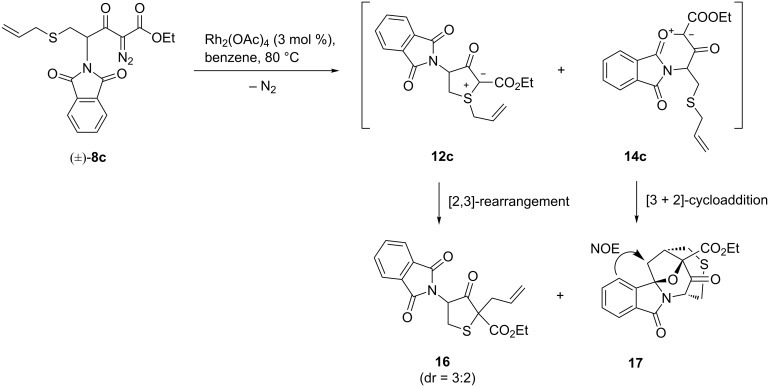
Rh(II)-catalysed carbenoid reactions of diazoester **8c**.

A different product pattern results from the rhodium(II)-catalysed dediazoniation of *S*-allyl-substituted diazoester **8c** (Scheme 6). In this case, the simultaneous formation of five-ring sulfonium ylide **12c** and six-ring carbonyl ylide **14c** is immediately followed by intramolecular pericyclic reactions with participation of the allylic π-system. Sulfonium ylide **12c** rearranges by the expected [2,3]-sigmatropic rearrangement [3,10,16] to form tetrahydrothiophene **16** (31% yield, two diastereomers), and carbonyl ylide **14c** is trapped by an intramolecular [3 + 2]-cycloaddition to give the pentacyclic compound **17** (35% yield). The regioselectivity of the latter reaction was established by NMR studies: after assignment of proton resonances from HMBC spectra, a NOESY NMR experiment indicated the correlation shown in Scheme 6. Intramolecular [3 + 2]-cycloaddition reactions of a carbonyl ylide with a nonactivated but suitably positioned olefinic bond have been known for some time [3,27–35] and can be used for the construction of multicyclic molecular frameworks (see also those carbonyl ylides that are part of an isomünchnone ring system [36–40]). In almost all cases encountered so far, the olefinic dipolarophile was tethered directly to a terminus of the carbonyl ylide dipole, and therefore the cycloaddition step gave rise to an annelated bicyclic substructure. In contrast, in our case as well as in [29], the olefinic dipolarophile is found as a substituent at a remote ring position of the cyclic carbonyl ylide, such that the intramolecular 1,3-dipolar cycloaddition generates a bridged oxabicyclo[3.2.1]octane substructure.

### Trapping of carbonyl ylides with dipolarophiles

**Scheme 7:**
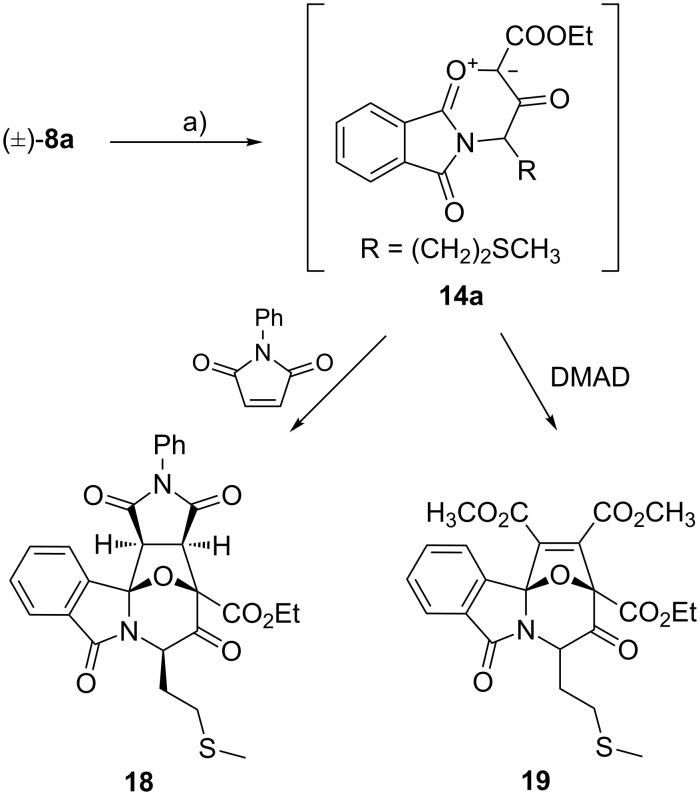
Tandem cyclisation/intermolecular cycloaddition of diazoester **8a**. Conditions: (a) Rh_2_(OAc)_4_ (3 mol %), *N*-phenylmaleimide or DMAD (1 equiv), benzene, 80 °C, 4 h.

In situ generated carbonyl ylides can be intercepted not only in intramolecular (as discussed above) but also in intermolecular [3 + 2] cycloaddition reactions (see, for example, [3,18–20,41–42]). By analogy with other 2-diazo-3-oxo-4-phthalimidocarboxylic esters [18–19], treatment of diazoester **8a** with catalytic Rh_2_(OAc)_4_ in the presence of an equimolar amount of *N*-phenylmaleimide (NPI) or dimethyl acetylenedicarboxylate (DMAD) produced the cycloaddition products **18** and **19**, respectively (Scheme 7). The signal sets of sulfonium ylides **12aA** and **12aB** were absent from the ^1^H NMR spectra of the crude product mixture. For adduct **18**, only one diastereomer was obtained (38% yield after workup), which, according to the observed NOE effects, has the pyrrolidinedione ring and the (CH_2_)_2_SCH_3_ chain in *exo* positions. This means that the cycloaddition has gone through an *exo* transition state with the dipolarophile approaching the carbonyl ylide from the face opposite to the sulfur-containing alkyl chain. In contrast, the NPI adducts of analogous carbonyl ylides derived from norleucine and alanine (**14**, R = *n*-C_4_H_9_ and CH_3_, respectively), were obtained as two diastereomers (the NCHR epimers) in approximately equal ratio [18]. The DMAD adduct **19** was also formed as a 2:1 mixture of two diastereomers; unfortunately, the highly viscous oil obtained could not be purified completely.

### Rhodium-catalysed carbenoid reactions of other mercapto-functionalised diazoesters

**Scheme 8:**
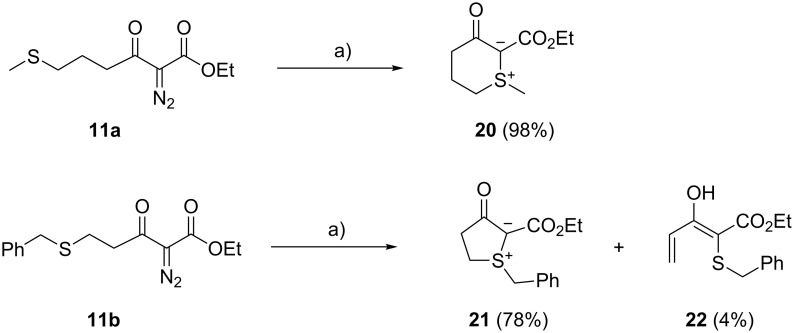
Carbenoid formation of sulfonium ylides from diazoesters **11a,b**. Conditions: (a) Rh_2_(OAc)_4_ (3 mol %), benzene, 80 °C, 2 h (**11a**) or 30 min (**11b**).

With respect to sulfonium ylide formation, aminoacid-derived diazoesters **8a**–**c** behave like the analogous mercapto-functionalised diazoesters that are devoid of the phthalimido group (Scheme 8). Thus, rhodium(II)-catalysed decomposition of 2-diazo-6-methylthio-3-oxohexanoate **11a** gave the stable six-ring sulfonium ylide **20** in practically quantitative yield. It has recently been reported that ruthenium(II) porphyrins are suitable catalysts for carbenoid sulfonium ylide formation as well [43]. In continuation of our comparative studies of dirhodium(II,II) tetracarboxylate and tetracarbonyldiruthenium(I,I) complexes [44], we found that the saccharinato complex [Ru_2_(CO)_5_[μ-sac)_2_]_2_ [45], the acetato complex [Ru_2_(CO)_4_(μ-OAc)_2_]_n_ and the trinuclear ruthenium(0) complex Ru_3_(CO)_12_ (3 mol % of catalyst in each case) gave yields of 74, 69, and 49% for the conversion of **11a** to **20**.

The rhodium-catalysed formation of a stable five-ring sulfonium ylide from the methyl ester analogue of diazoester **11b** has previously been reported [15]. We confirm these results with **11b**; however, in addition to sulfonium ylide **21** we also isolated the dienol **22** as a minor byproduct. The latter compound likely results from a thermally induced ring-opening β-elimination reaction of ylide **21**, as was previously reported for other cyclic [14] and acyclic [46] sulfonium ylides.

## Conclusion

Metal-carbene intermediates derived from α-diazoesters and α-diazo-β-ketoesters are known for their high reactivity towards a range of functional groups. It is therefore interesting to gather information about the chemoselectivity of these reactive intermediates. For intramolecular reactions, the ring size of the products can make an additional contribution to the observed chemoselectivity. In this study, we have addressed the competition between two intramolecular ylide-forming pathways, namely the formation of cyclic sulfonium and carbonyl ylides. To this end, 2-diazo-3-oxo-4-phthalimidocarboxylic esters **8a**–**c** were prepared from the mercapto-functionalised aminoacids methionine, *S*-benzylcysteine, and *S*-allylcysteine. Rhodium(II)-catalysed decomposition of the diazoesters was found to produce both six- or five-membered cyclic sulfonium ylides and six-membered carbonyl ylides. In a qualitative manner, it can be stated that in the case of **8a** formation of the six-membered cyclic carbonyl ylide **14a** clearly supersedes the formation of the six-membered sulfonium ylide **12a**, although the structurally analogous cyclic sulfonium ylide **20** is formed quantitatively from diazoester **11a**, which bears no phthalimido group. This result is confirmed by the absence of **12a** from the reactions in which the carbonyl ylide was intercepted by intermolecular [3 + 2] cycloaddition with the dipolarophiles NPI and DMAD (notice that carbonyl ylide dimer **13a** also results from an intermolecular cycloaddition reaction). With the cysteine-derived diazoesters **8b** and **c**, on the other hand, it appears that the formation of the five-membered cyclic sulfonium ylides **12b** and **c** and of the six-membered cyclic carbonyl ylides **14b** and **c** is about equally efficient. Interestingly, the *S*-allyl group spontaneously transforms both types of ylides into non-ylidic products, namely by [2,3]-sigmatropic rearrangement for the S-ylide and intramolecular [3 + 2]-cycloaddition for the carbonyl ylide.

## Supporting Information

The supporting information contains experimental procedures and characterisation details for the synthesised compounds.

File 1 Experimental part.
