# Supplementary material for: Intramolecular carbenoid ylide forming reactions of 2-diazo-3-keto-4-phthalimidocarboxylic esters derived from methionine and cysteine
Source: Beilstein J Org Chem. 2012 Mar 22;8:433–40. doi: 10.3762/bjoc.8.49 (PMC3326622; doi:10.3762/bjoc.8.49)
Supplement: File 1 — Experimental part. [file Beilstein_J_Org_Chem-08-433-s001.pdf]

Supporting Information

for

Intramolecular carbenoid ylide forming reactions of 2-diazo-3-oxo-4-phthalimidocarboxylic esters derived from methionine and cysteine

Marc Enßle, Stefan Buck, Roland Werz and Gerhard Maas*

Address: Institute of Organic Chemistry I, University of Ulm, Albert-Einstein-Allee 11, D-89081 Ulm, Germany

Email: Gerhard Maas - gerhard.maas@uni-ulm.de

* Corresponding author

Experimental part

General information. ^{1}H and ^{13}C NMR spectra were recorded on a Bruker Avance 400 spectrometer (^{1}H: 400.13 MHz; ^{13}C: 100.62 MHz); δ values are reported in ppm and coupling constants are expressed in Hertz (Hz). The signal of the solvent ($CDCl_3$, CD_3CN or DMSO-d_6) was used as the internal standard; δ values are reported in ppm. When necessary, ^{13}C signal assignments were derived from C,H COSY, HSQC and HMBC spectra. IR spectra: Bruker Vector 22; wavenumbers [cm^{-1}] are given. Elemental analyses: Elementar Vario Micro Cube. Mass spectra: Finnigan MAT SSQ 700 (CI spectra, 70–100 eV, methane as reagent gas) and Bruker Daltonics micrOTOFQ (HRMS (+)-ESI spectra). Column chromatography was performed under hydrostatic pressure (silica gel Si 60, Macherey–Nagel, 0.063–0.2 mm). Melting points were determined with a Büchi Melting Point B-540 apparatus.

Analytical HPLC for determination of enantiomeric ratios: Varian ProStar 210 station with a diode-array detector Varian PDA 330 and a Varian Star chromatography workstation 5.5; column: phenomenex Lux 1 and Lux 2, 250 × 4.6 mm; elution with 2-propanol/*n*-hexane (2:8 v/v), flow 1 mL/min; in one case, a β-cyclodextrin column (astec Cyclobond I (β) 5 μm, 250×4.6 mm) was used. Polarimetry: Perkin-Elmer polarimeter 231. The reactions were carried out in air unless otherwise noted. Column chromatography was performed under hydrostatic pressure (silica gel Si 60, Macherey-Nagel, 0.063–0.2 mm).

Materials. L-Methionine and DL-methionine were purchased from Merck KGaA. *S*-Benzyl-L-cysteine [1], *S*-allyl-L-cysteine [2], 4-(methylsulfanyl)butyric acid [3], 3-(benzylsulfanyl)propionic acid [4], and imidazole-1-sulfonylazide hydrochloride [5] were prepared by following published procedures.

***N,N*-Phthaloylamino acids 6a–c:** They were prepared from the corresponding aminoacids **5a–c**, phthalic anhydride (1 equiv) and triethylamine (10 mol %) in toluene under reflux as reported for *N*-phthaloyl-L-phenylalanine [6,7].

***N,N*-Phthaloyl-L-methionine (6a)**: Yield: 97%; recrystallisation from diisopropyl ether/petroleum ether, colourless solid, mp 121–126 °C (lit. [8]: 124 °C); $[\alpha]_{589}^{20}$ = −43.5 °(c = 1 in MeOH) (lit [9]: $[\alpha]_{589}^{20}$ = −44.1 ° (*c* = 1 in MeOH)). ^{1}H NMR ($CDCl_3$): δ = 2.05 (s, 3 H, SCH_3), 2.45–2.57 (m, 4 H, $(CH_2)_2$), 5.16–5.17 (m, 1 H, H_{Pht}), 7.71–7.73 (m, 2 H_{Pht}), 7.84–7.86 (m, 2 H, H_{Pht}), 10.90 (bs, 1 H, COOH) ppm. ^{13}C NMR ($CDCl_3$): δ = 15.33 (SCH_3), 27.87 (SCH_2), 30.87 (SCH_2CH_2), 50.73 (*C*HCO), 123.77 (CH_{Ar}), 131.75 (COC_{Ar}), 134.43 (CH_{Ar}), 167.66 (NC=O), 175.03 (COOH) ppm. Anal. calcd. for $C_{13}H_{13}NO_4S$ (279.32): C, 55.90; H, 4.69; N, 5.01; found: C, 56.04; H, 4.68; N, 5.07.

***S*-Benzyl-*N,N*-phthaloyl-L-cysteine (6b)**: Yield: 75%; recrystallisation from toluene, colourless solid, mp 122–124 °C (lit. [10]: 128–129 °C); $[\alpha]_{589}^{20}$ = −109.1° (*c* = 0.9 in MeOH) (lit. [S10]: $[\alpha]_{589}^{20}$ = −150.1° (*c* = 1.1 in MeOH). In this reference, it is also mentioned that partial racemisation was observed when the synthesis was conducted at 135−140 °C rather than 110−115 °C, resulting in a drop of [α] to −67 to −85°. While we could confirm that **6b** was configurationally stable when kept in boiling toluene for one hour, we did not investigate further the reason for the lower optical rotation of our sample as compared to lit. [S10], because we were aware of the complete racemisation during conversion of **6b** into ketoester **7b**.] ^{1}H NMR ($CDCl_3$): δ = 3.22/3.31 (AB part of ABX system, 2 H, ^3J = 11.2 and 4.9 Hz, ^2J = 14.5 Hz, $NCHCH_2$), 3.73/3.74 (AB system, *J* = 13.5 Hz, 2 H, $PhCH_2$), 5.03 (X part of ABX system, ^3J = 11.2 and 4.9 Hz, 1 H, NCH), 7.22–7.32 (m, 5 H_{Ph}), 7.76–7.78 (m, 2 H, H_{Pht}),

7.89–7.91 (m, 2 H, H_{Pht}), 9.00 (very broad signal, 1 H, COOH) ppm. ^{13}C NMR ($CDCl_3$): δ = 29.95 (S*C*H_2CH), 35.75 (S*C*H_2Ph), 50.76 (NCH), 123.89, 127.42, 128.79, 129.07, 131.78 (NCO*C*$_{Pht}$), 134.47, 137.23, 167.53 (NC=O), 173.39 (COOH) ppm. Anal. calcd. for $C_{18}H_{15}NO_4S$ (341.39): C, 63.33; H, 4.43; N, 4.10; found: C, 63.26; H, 4.43; N, 4.20.

***S*-Allyl-*N,N*-phthaloyl-L-cysteine (6c):** Yield: 93%; brown oil, which could not be purified completely. IR (film): ν = 3421 br s (COOH), 2924 w, 1717 s (C=O), 1393 m, 719 w cm^{-1}. ^1H NMR ($CDCl_3$): δ = 3.10/3.20 (AB part of ABX system, ^3J = 8.0 and 6.5 Hz, ^2J = 13.8 Hz, 1 H, SCH_2CH=), 3.34/3.36 (m, AB part of ABX, 2 H, NCHCH_2), 5.00 (X part of ABX system, ^3J = 9.8 and 6.2 Hz, 1 H, NCH), 5.13–5.18 (m, 2 H, CH_2=), 5.69–5.80 (m, 1 H, CH=), 7.73–7.77 (m, 2 H, H_{Pht}), 7.85–7.90 (m, 2 H, H_{Pht}) ppm. ^{13}C NMR ($CDCl_3$): δ = 29.19 (*C*H_2CHN), 34.32 (S*C*H_2CH=), 50.85 (NCH), 118.27 (*C*H_2=CH), 123.87 (CH_{Pht}), 131.76 (NCO*C*$_{Pht}$), 133.56 (CH_2=*C*H), 134.47 (CH_{Ar}), 167.57 (NC=O), 173.65 (COOH) ppm. MS (CI): *m*/*z* (%) = 320 (22), 292 (100, $[M]^+$), 246 (57).

Synthesis of β-ketoesters 7 and 10

General Procedure: The appropriate *N*-phthaloylaminoacid **6** or carboxylic acid **9** (1 equiv) was dissolved in dry THF (100–200 mL). *N*,*N*'-Carbonyldiimidazole (1.1 equiv) was added in portions, and the mixture was stirred for 16 h under argon at room temperature. In a separate flask purged with argon and cooled in an ice bath, a solution of malonic acid monoethyl ester (1.1 equiv) in dry THF (100–200 mL) was prepared. To the stirred solution were added anhydrous $MgCl_2$ (0.6 equiv) and triethylamine (1.2 equiv, gradual addition so as to keep the temperature below 10 °C), and the formed white slurry was stirred for 1 h at 0 °C. The ice bath was removed and the two mixtures were combined and stirred for 3.5 h at room temperature. Most of the solvent (90%) was evaporated, and diethyl ether and water (60–100 mL each) were added. The phases were separated, and the water phase was extracted with three portions (3 × 15–20 mL) of ether. The combined organic phases were extracted with three portions of water (3 × 15–20 mL) and once with brine. After drying with Na_2SO_4 the solvent was removed, and the crude product was purified by column chromatography (eluent: ethyl acetate–cyclohexane (4:1 for **7a**–**c**, 1:5 for **10a**,**b**)). The eluent was evaporated, and the last traces of solvent were removed at 25 °C/10^{-3} bar.

6-Methylsulfanyl-3-oxo-4-phthalimidohexanoic acid ethyl ester (7a): Prepared from **6a** (12.22 g, 43.75 mmol). Colourless solid (4.46 g, 12.76 mmol, 30% yield), mp 60–63 °C, enantiomeric ratio = 1.1:1 by HPLC (Lux 1), $[\alpha]_{589}^{20}$ = −9.4 (c = 1 in MeOH). IR (KBr): ν =

2976 w, 2918 w, 1776 m, 1746 s, 1715 s, 1383 s cm^{-1}. ^{1}H NMR ($CDCl_3$): keto form (97%): δ = 1.20 (t, ^{3}J = 7.1 Hz, 3 H, CH_2CH_3), 2.05 (s, 3 H, SCH_3), 2.37–2.55 (m, 4 H, SCH_2CH_2), 3.47/3.52 (AB quartet, ^{2}J = 15.7 Hz, 2 H, CH_2COOEt), 4.12/4.13 (2 q, ^{3}J = 7.1/7.2 Hz, 2 H, OCH_2), 5.09–5.13 (m, 1 H, NCH), 7.75–7.77 (m, 2 H, H_{Pht}), 7.87–7.89 (m, 2 H, H_{Pht}); enol form (3%): 5.16 (s, 1H, C=C*H*CO), 12.31 (s, 1H, *H*OC=C) ppm. ^{13}C NMR ($CDCl_3$): δ = 14.08, 15.52, 27.15, 30.90, 46.42, 57.73, 61.82, 123.87, 131.76, 134.63, 166.38, 167.79, 197.56 ppm. MS (CI): *m*/*z* (%) = 378 (10), 350 (69, $[M]^+$), 304 (100). Anal. calcd. for $C_{17}H_{19}NO_5S$ (349.41): C, 58.44; H, 5.48; N, 4.01; found: C, 58.31; H, 5.50; N, 4.16.

5-Benzylsulfanyl-3-oxo-4-phthalimidopentanoic acid ethyl ester (7b): Prepared from **6b** (7.00 g, 20.51 mmol). Yellow oil (5.10 g, 12.39 mmol, 60% yield), enantiomeric ratio = 51:49 by HPLC (Lux 2), $[\alpha]_{589}^{20}$ = 0.1 (c = 1 in MeOH). IR (film): *ν* = 3061, 3028, 2982, 2932 (all w), 1776 m, 1723 s, 1384 s, 720 m cm^{-1}. ^{1}H NMR ($CDCl_3$): keto form (94%): δ = 1.21 (t, ^{3}J = 7.1 Hz, 3 H, CH_2CH_3), 3.12/3.24 (AB part of ABX system, ^{3}J = 10.9 and 4.8 Hz, ^{2}J = 14.4 Hz, 2 H, SCH_2CH), 3.42/3.50 (AB system, ^{2}J = 16.0/15.9 Hz, 2 H, CH_2CO_2Et), 3.70 (s, 2 H, SCH_2Ph), 4.11/4.12 (2 q, *J* = 7.1/7.2 Hz, 2 H, OCH_2CH_3), 5.03 (X part of ABX system, *J* = 10.8 and 4.8 Hz, 1 H, NCH), 7.20–7.31 (m, 5 H, H_{Ph}), 7.77–7.79 (m, 2 H, H_{Pht}), 7.89–7.91 (m, 2 H, H_{Pht}); enol form (6%): 5.12 (s, 1H, C=C*H*CO), 12.29 (s, 1H, *H*OC=C) ppm. ^{13}C NMR ($CDCl_3$): δ = 14.10, 29.51, 35.93, 46.77, 57.28, 61.88, 123.99, 127.42, 128.78, 129.08, 131.78, 134.65, 137.28, 166.30, 167.70, 197.28 ppm. MS (CI): *m*/*z* (%) = 440 (21), 412 (86, $[M]^+$), 366 (100), 288 (32), 91 (48). Anal. calcd. for $C_{22}H_{21}NO_5S$ (411.48): C, 64.22; H, 5.14; N, 3.40; found: C, 64.16; H, 5.07; N, 3.33.

5-Allylsulfanyl-3-oxo-4-phthalimidopentanoic acid ethyl ester (7c): Prepared from **6c** (13.75 g, 47.20 mmol). Yellow oil (3.19 g, 8.83 mmol, 19% yield). IR (film): *ν* = 2986, 2930, 2885 (all w), 1777 m, 1742 s, 1717 s, 1388 s, 720 m cm^{-1}. ^{1}H NMR ($CDCl_3$): keto form (92%): δ = 1.22 (t, ^{3}J = 7.1 Hz, 3 H, OCH_2CH_3), 3.04–3.20 (m, 3 H, SCH_2CH= and $NCHCH^A$), 3.35 (B part of ABX system, ^{3}J = 4.8 Hz, ^{2}J = 14.5 Hz, 1 H, $NCHCH^B$), 3.47/3.54 (AB quartet, ^{2}J = 15.7 Hz, 2 H, CH_2CO_2Et), 4.11/4.12 (2 q, ^{3}J = 7.1/7.2 Hz, 2 H, OCH_2), 4.99 (X part of ABX system, ^{3}J = 10.8 and 4.8 Hz, 1 H, NCH), 5.11–5.19 (m, 2 H, CH_2=CH), 5.68–5.78 (m, 1 H, CH_2=C*H*), 7.77–7.79 (m, 2 H_{Pht}), 7.89–7.91 (m, 2 H, H_{Pht}); enol form (8%): 12.31 (s, 1H, *H*OC=C) ppm. ^{13}C NMR ($CDCl_3$): δ = 14.09, 28.79, 34.47, 46.82, 57.31, 61.87, 118.17, 123.97, 131.75, 133.66, 134.66, 166.29, 167.71, 197.36. MS (CI): *m*/*z* (%) = 390 (26), 362 (100, $[M]^+$), 316 (84), 288 (18) ppm. Anal. calcd. for $C_{18}H_{19}NO_5S$ (361.42): C, 59.82; H, 5.30; N, 3.88; found: C, 59.73; H, 5.25; N, 3.82.

6-Methylsulfanyl-3-oxohexanoic acid ethyl ester (10a): Prepared from 4-methylsulfanylbutanoic acid (**9a**, 3.26 g, 24.29 mmol). Yellowish oil (1.37 g, 6.71 mmol, 27% yield). IR (film): ν = 2981 s, 2918 s, 1743 s, 1715 m, 1410 m, 1367 m cm^{-1}. ^{1}H NMR ($CDCl_3$): keto form (94%): δ = 1.28 (t, *J* = 7.2 Hz, 3 H, OCH_2CH_3), 1.91 (quin, 2 H, SCH_2CH_2), 2.07 (s, 3 H, SCH_3), 2.51 (t, *J* = 7.0 Hz, 2 H, SCH_2), 2.69 (t, *J* = 7.1 Hz, 2 H, CH_2CH_2CO), 3.45 (s, 2 H, CH_2CO_2Et), 4.20 (q, *J* = 7.1 Hz, 2 H, OCH_2); enol form (6%): 5.00 (s, 1H, C=C*H*CO), 12.11 (s, 1H, *H*OC=C) ppm. ^{13}C NMR ($CDCl_3$): δ = 14.26, 15.33, 22.51, 33.49, 41.48, 49.60, 61.55, 167.28, 202.30 ppm. MS (CI): *m*/*z* (%) = 205 (29, $[M]^+$ for $C_9H_{16}O_3S$), 159 (100), 157 (78).

5-Benzylsulfanyl-3-oxopentanoic acid ethyl ester (10b): Prepared from 3-benzylsulfanylpropanoic acid (**9b**, 17.60 g, 89.67 mmol). Yellowish oil (12.70 g, 47.68 mmol, 53% yield). IR (film): ν = 3060 w, 3026 w, 2925 w, 1733 s cm^{-1}. ^{1}H NMR ($CDCl_3$): keto form (92%): δ = 1.27 (t, *J* = 7.1 Hz, 3 H, OCH_2CH_3), 2.67 (t, 2 H), 2.76 (t, 2 H), 3.39 (s, 2 H, CH_2CO_2Et), 3.72 (s, 2 H, $PhCH_2$), 4.19 (q, *J* = 7.1 Hz, 2 H, OCH_2), 7.22–7.32 (m, 5 H, H_{Ph}); enol form (8%): 4.97 (s, 1H, C=C*H*CO), 12.06 (s, 1H, *H*OC=C) ppm. ^{13}C NMR ($CDCl_3$): δ = 14.25, 25.11, 36.88, 42.95, 49.52, 61.60, 127.26, 128.72, 128.97, 138.29, 167.02, 201.09 ppm. Anal. calcd. for $C_{14}H_{18}O_3S$ (266.36): C, 63.13; H, 6.81; found: C, 63.16; H, 6.81.

Synthesis of α-diazo-β-ketoesters 8 and 11

2-Diazo-6-methylsulfanyl-3-oxo-4-phthalimidohexanoic acid ethyl ester (8a), method A: β-Ketoester **7a** (1.00 g, 2.86 mmol) was dissolved in dry acetonitrile (20 mL) and *p*-toluenesulfonyl azide (0.62 g, 3.15 mmol) as well as triethylamine (0.41 mL, 5.72 mmol) were added. The solution was stirred at room temperature for 16 h, and then the solvent was replaced by CH_2Cl_2. Upon addition of *n*-pentane, the byproduct, *p*-toluenesulfonyl amide, was precipitated and was filtered off. The mother liquor was evaporated to dryness, and the residue was purified by column chromatography with ethyl acetate–cyclohexane (1:4) as eluent. After evaporation of the solvents (last traces at 25 °C/10^{-3} bar), a yellow oil was obtained (0.66 g, 1.76 mmol, 61%); $[\alpha]_{589}^{20}$ = 0.3 (*c* = 1 in MeOH). IR (film): ν = 2981 s, 2919 s, 2144 s ($C=N_2$), 1777 m, 1730 s, 1714 m, 1468 m, 1441 m, 1384 s, 746 m, 719 s cm^{-1}. ^{1}H NMR ($CDCl_3$): δ = 1.30 (t, *J* = 7.1 Hz, 3 H, OCH_2CH_3), 2.10 (s, 3 H, SCH_3), 2.33–2.41 (m, 1 H, SCH_2CH^A), 2.58 (t, 2 H, SCH_2), 2.72–2.81 (m, 1 H, SCH_2CH^B), 4.30 (q, *J* = 7.1 Hz, 2 H, OCH_2), 5.71 (dd, *J* = 10.5 and 4.2 Hz, 1 H, NCH), 7.71–7.73 (m, 2 H_{Pht}), 7.84–7.86 (m, 2 H, H_{Pht}) ppm. ^{13}C NMR ($CDCl_3$): δ = 14.45, 15.51, 27.56, 31.43, 57.15, 62.98, 76.14, 123.63, 131.98, 134.27, 160.69, 168.29, 187.51 ppm. MS (CI): *m*/*z* (%) = 404 (5), 376 (26, $[M]^+$), 348

(100), 302 (2). Anal. calcd. for $C_{17}H_{17}N_3O_5S$ (375.40): C, 54.39; H, 4.56; N, 11.19; found: C, 54.34; H, 4.66; N, 10.90.

Method B: Thionyl chloride (13 mL, 180 mmol) was added from a dropping funnel to a solution of *N*-phthaloyl-L-methionine (**6a**) (6.83 g, 24.45 mmol) in dry dichloromethane (100 mL). After completed addition, the reaction mixture was heated under reflux for 2 h, the solvent was removed in vacuo and the residual volatiles at 25 °C/10^{-3} mbar. The acid chloride was obtained as a brown oil, which was combined with ethyl diazoacetate (5.4 mL, 51.1 mmol) in a round-bottom flask protected from light. The mixture was stirred at ambient temperature for four days, and then the volatiles (ethyl chloroacetate and residual ethyl diazoacetate) were evaporated at 25 °C/10^{-3} mbar. The residual oil was submitted to column chromatography (silica gel (480 g), cyclohexane–ethyl acetate (6:4) as eluent) to furnish diazoester **8a** as a yellow oil; yield: 2.60 g (6.92 mmol, 28% based on **6a**). Enantiomeric ratio = 83:17 (HPLC, β-cyclodextrin column, eluent water–methanol (55:45)).

5-Benzylsulfanyl-2-diazo-3-oxo-4-phthalimidopentanoic acid ethyl ester (8b): β-Ketoester **7b** (2.55 g, 6.20 mmol) was dissolved in dry CH_2Cl_2 (60 mL). Imidazole-1-sulfonylazide hydrochloride (1.56 g, 7.40 mmol) and triethylamine (4.30 mL, 31.00 mmol) were added. The solution was stirred at 40 °C for 16 h. After cooling, the solution was diluted with CH_2Cl_2 (30 mL) and extracted twice with 1 M aqueous HCl and once with water, then dried (Na_2SO_4). After evaporation of the solvent, the crude product was purified by column chromatography with ethyl acetate–cyclohexane (1:1) as eluent. The product was obtained as a solid (2.68 g, 6.13 mmol, 99% yield), mp 90–95 °C, $[\alpha]_{589}^{20} = -0.3$ (c = 1 in MeOH), enantiomeric ratio = 1.1:1 by HPLC (Lux 1). IR (film): ν = 3062, 3029, 2982, 2930 (all w), 2143 s (C=N_2), 1776 s, 1715 s, 1468 w, 1385 s, 717 s cm^{-1}. ^1H NMR ($CDCl_3$): δ = 1.30 (t, ^3J = 7.1 Hz, 3 H, OCH_2CH_3), 3.27/3.43 (AB part of ABX system, ^2J = 13.9 Hz, $^3J^{AX}$ = 4.9 Hz, $^3J^{BX}$ = 10.5 Hz, 2 H, SCH_2CH), 3.83 (AB quartet, ^2J = 13.2 Hz, 2 H, $PhCH_2S$), 4.28 (virtual q, J = 7.1 Hz, 2 H, OCH_2), 5.77 (X part of ABX system, ^3J = 10.5 and 4.9 Hz, 1 H, NCH), 7.16–7.30 (m, 1 H, H_{Ph}), 7.23–7.27 (m, 2 H, H_{Ph}), 7.32–7.34 (m, 2 H, H_{Ph}), 7.71–7.75 (m, 2 H, H_{Pht}), 7.85–7.89 (m, 2 H, H_{Pht}) ppm. ^{13}C NMR ($CDCl_3$): δ = 14.28, 30.14, 36.02, 56.50, 61.93, 76.24, 123.52, 127.08, 128.38, 128.99, 131.72, 134.07, 137.98, 160.44, 167.82, 186.14 ppm. MS (CI): m/z (%) = 466 (19), 438 (86, $[M]^+$), 410 (100). Anal. calcd. for $C_{22}H_{19}N_3O_5S$ (437.47): C, 60.40; H, 4.38; N, 9.61; found: C, 60.54; H, 4.40; N, 9.52.

5-Allylsulfanyl-2-diazo-3-oxo-4-phthalimidopentanoic acid ethyl ester (8c): Prepared from β-ketoester **7c** (1.00 g, 2.77 mmol) as described above for **8b**. Yellow oil (0.94 g, 2.43

mmol, 88% yield), enantiomeric ratio = 1.4:1 by HPLC (Lux 1). IR (film): ν = 2993, 2939, 2921 (all w), 2150 s ($C=N_2$), 1775 s, 1737 s, 1389 s, 718 s cm^{-1}. ^1H NMR ($CDCl_3$): δ = 1.29 (t, ^3J = 7.1 Hz, 3 H, OCH_2CH_3), 3.15–3.20 (m, 1 H, $SCH^AHCH=$), 3.25–3.30 (m, 3 H, $SCHH^BCH=$ and SCH^AHCHN), 3.40 (B part of ABX system, ^2J = 13.9 Hz, $^3J^{BX}$ = 10.4 Hz, 1 H, $SCHH^BCHN$), 4.28 (q, J = 7.1 Hz, 2 H, OCH_2), 5.06–5.08 (m, ^3J = 10.0 Hz, 1 H of $CH_2=CH$), 5.19 (m, ^3J = 16.1 Hz, 1 H of $CH_2=CH$), 5.70 (X part of ABX system, ^3J = 10.4 and 5.1 Hz, 1 H, NCH), 5.74–5.84 (m, 1 H, $CH_2=CH$), 7.71–7.73 (m, 2 H, H_{Pht}), 7.85–7.87 (m, 2 H, H_{Pht}) ppm. ^{13}C NMR ($CDCl_3$): δ = 14.42, 29.45, 34.64, 56.67, 62.06, 76.39, 117.68, 123.66, 131.93, 134.23, 134.25, 160.57, 167.97, 186.33 ppm. MS (CI): *m*/*z* (%) = 416 (13), 388 (69, $[M]^+$), 360 (100), 314 (18), 246 (27). Anal. calcd. for $C_{18}H_{17}N_3O_5S$ (387.41): C, 55.81; H, 4.42; N, 10.85; found: C, 55.79; H, 4.45; N, 10.85.

2-Diazo-6-methylsulfanyl-3-oxohexanoic acid ethyl ester (11a): Prepared from β-ketoester **7a** (1.32 g, 6.46 mmol) and *p*-toluenesulfonyl azide (1.40 g, 7.10 mmol) as described above for **8a**. Column chromatography with ethyl acetate–cyclohexane (1:4) furnished a yellow oil (0.82 g, 3.56 mmol, 55% yield). IR (film): ν = 2985 s, 2920 s, 2137 s ($C=N_2$), 1793 s, 1715 m, 1375 m, 733 s cm^{-1}. ^1H NMR ($CDCl_3$): δ = 1.33 (t, J = 7.1 Hz, 3 H, OCH_2CH_3), 1.95 (m, 2 H, SCH_2CH_2), 2.09 (s, 3 H, SCH_3), 2.55 (t, J = 7.2 Hz, 2 H, SCH_2), 2.98 (t, J = 7.2 Hz, 2 H, CH_2CO), 4.30 (q, J = 7.1 Hz, 2 H, OCH_2) ppm. ^{13}C NMR ($CDCl_3$): δ = 14.51, 15.45, 23.61, 33.76, 39.12, 61.59, 76.12, 161.49, 192.34 ppm. Anal. calcd. for $C_9H_{14}N_2O_3S$ (230.29): C, 46.94; H, 6.13; N, 12.16; found: C, 47.06; H, 6.08; N, 12.02.

5-Benzylsulfanyl-2-diazo-3-oxopentanoic acid ethyl ester (11b): Prepared from β-ketoester **10b** (12.70 g, 47.68 mmol) and imidazole-1-sulfonylazide hydrochloride (12.00 g, 57.30 mmol) as described for **8b**. Column chromatography with ethyl acetate–cyclohexane (1:6) furnished a yellow oil (11.40 g, 38.99 mmol, 82% yield). IR (film): ν = 3028 w, 2983 m, 2929 m, 2136 s ($C=N_2$), 1719 s, 1373 m cm^{-1}. ^1H NMR ($CDCl_3$): δ = 1.32 (t, ^3J = 7.1 Hz, 3 H, OCH_2CH_3), 2.72 (t, J = 7.2 Hz, 2 H, SCH_2CH_2), 3.12 (t, J = 7.2 Hz, 2 H, CH_2CO), 3.74 (s, 2 H, $PhCH_2S$), 4.29 (q, J = 7.1 Hz, 2 H, OCH_2), 7.20–7.34 (m, 5 H, H_{Ph}) ppm. ^{13}C NMR ($CDCl_3$): δ = 14.44, 25.68, 36.46, 40.01, 61.58, 76.31, 127.06, 128.56, 128.96, 138.30, 161.27, 190.92 ppm. Anal. calcd. for $C_{14}H_{16}N_2O_3S$ (292.36): C, 57.52; H, 5.52; N, 9.58; found: C, 57.59; H, 5.65; N, 9.56.

Catalytic decomposition of the γ-phthalimido-α-diazo-β-ketoesters

Decomposition of *rac*-2-diazo-6-methylsulfanyl-3-oxo-4-phthalimidohexanoic acid ethyl ester ((±)-8a): To a suspension of $Rh_2(OAc)_4$ (13 mg, 0.03 mmol) in dry benzene (20 mL)

under reflux was added a solution of diazoester (±)-**8a** (0.376 g, 1.00 mmol) in dry benzene (5 mL) through a syringe pump over a period of 1 h. After 0.5 h the diazo compound had been consumed (IR control, disappearance of the ν(CN_2) absorption). The solvent was evaporated (last traces at 25 °C/10^{-3} mbar) and the resulting redish solid was triturated with ethyl acetate. The insoluble off-white residue, sulfur ylide **12a**, was filtered off and dried at 25 °C/10^{-3} mbar. The filtrate contained the carbonyl ylide dimer **13a**, which was purified further by flash column chromatography (20 g of silica gel 60, eluent cyclohexane–ethyl acetate (1:1)). The solvent was evaporated to leave **13a** as a yellow solid, which still contained some impurities but due to its limited stability could not be purified further.

1-Methyl-3-oxo-4-phthalimido-3,4,5,6-tetrahydro-1λ^4-thiapyran-2-carboxylic acid ethyl ester (12a): Yield: 0.106 g (0.31 mmol, 31%), 4:1 mixture of two diastereomers, mp 236–238 °C (dec.). IR (KBr): ν = 2977, 2949, 2900 (all w), 1708 s, 1619 m, 1572 s, 1387 s, 743 s cm^{-1}. ^{1}H NMR ([D$_6$]DMSO), isomer **A** (major) and **B** (minor) : δ = 1.13 (t, J = 7.1 Hz, 2.4 H, OCH_2CH_3, **A**), 1.14 (t, J = 7.1 Hz, 0.6 H, OCH_2CH_3, **B**), 2.35–2.41 (dddd, ^2J = 14.1 Hz, ^3J = 5.7, 2.8, 2.8 Hz, 0.8 H, SCH_2CH^{eq}, **A**), 2.57–2.72 (m, 0.4 H, SCH_2CH_2, **B**), 2.80 (s, 2.4 H, SCH_3, **A**), 2.83 (s, 0.6 H, SCH_3, **B**), 3.05 (dddd, $^2J \approx {}^3J \approx$ 14.1 Hz, ^3J = 12.7, 2.9 Hz, 0.8 H, SCH_2CH^{ax}, **A**), 3.41–3.46 (m, 1 H, SCH^{eq} of **A** and **B**), 3.77–3.83 (m, 1 H, SCH^{ax} of **A** and **B**; for isomer **A**: td, $^2J \approx {}^3J \approx$ 14.1 Hz, ^3J = 2.7 Hz), 3.94–4.07 (m, 2 H, OCH_2, **A** and **B**), 4.65 (dd, J = 12.7 and 5.7 Hz, 0.8 H, NCHCO, **A**), 4.75 (dd, J = 9.1 and 5.6 Hz, 0.2 H, NCHCO, **B**), 7.80–7.96 (broadened "s", 4 H, H_{Pht}, **A** and **B**) ppm. ^{13}C NMR ([D$_6$]DMSO), isomer **A**: δ = 14.50, 22.06, 28.37, 32.17, 52.08, 58.54, 73.63, 122.95, 131.50, 134.48, 165.74, 167.77, 176.60 ppm. MS (CI): m/z (%) = 376 (36), 348 (93, [M]$^+$), 302 (67), 288 (100). Anal. calcd. for $C_{17}H_{17}NO_5S$ (347.39): C, 58.78; H, 4.93; N, 4.03; found: C, 57.52; H, 4.80; N, 4.15; $C_{17}H_{17}NO_5S$ (347.39) + 0.45 H_2O requires: C, 57.44; H, 5.08; N, 3.94.

Carbonyl ylide dimer 13a: Yield: 0.161 g (43%; the yield was determined on the crude product mixture by ^{1}H NMR using 1-chloro-4-nitrobenzene as internal standard). IR (KBr): ν = 3059 w, 2981 w, 2917 w, 1756 m, 1719 s, 1370 s, 759 w, 720 w cm^{-1}. ^{1}H NMR ($CDCl_3$): δ = 0.73 (t, ^3J = 7.1 Hz, 3 H, OCH_2CH_3), 1.88–1.96 (m, 1 H, SCH_2CH^A), 1.99 (s, 3 H, SCH_3), 2.11–2.29 (m, 2 H, SCH_2), 2.79–2.88 (m, 1 H, SCH_2CH^B), 3.34–3.39 and 3.65–3.70 (2 m, 1 H each, OCH_2), 4.60 (dd, ^3J = 10.7 and 3.8 Hz, 1 H, NCH), 7.53–7.91 (m, 4 H, H_{Pht}) ppm. ^{13}C NMR ($CDCl_3$): δ = 13.35, 15.16, 28.53, 29.17, 63.50, 64.61, 86.80, 94.13, 122.57, 123.89, 131.65, 133.03, 134.62, 141.85, 161.57, 166.33, 195.66 ppm. MS (CI): m/z (%) = 376 (11), 348 (83, [M]$^+$/2) (M = $C_{34}H_{34}N_2O_{10}S_2$).

Decomposition of 5-benzylsulfanyl-2-diazo-3-oxo-4-phthalimidopentanoic acid ethyl ester ((±)-8b)

According to the procedure described for the decomposition of **8a**, a redish solid was obtained, which contained sulfur ylide **12b** and dimer **13b** in a 1.45:1 molar ratio (^{1}H NMR integration; by integration against an added standard, yields of 36.9 (**12b**) and 25.3% (**13b**) were calculated). Trituration with ethyl acetate left an insoluble white residue, which was filtered off and dried at 25 °C/10^{-3} mbar. It was a mixture of **12b** and **13b**, which could be separated by trituration with methanol, since only **12b** was soluble in methanol.

1-Benzyl-4-(phthalimido)-3-oxo-4,5-dihydro-3*H*-1λ^4-thiophene-2-carboxylic acid ethyl ester (12b*)*: white solid, mixture of two diastereomers (3:1 ratio by ^{1}H NMR integration). ^{1}H NMR ($CDCl_3$), isomer **A** (major) and **B** (minor): δ = 1.33 (t, J = 7.1 Hz, 3 H, OCH_2CH_3, **A** and **B**), 3.30–3.36 (m, 1 H, 5-H^A, **A** and **B**), 3.59 (dd, J = 13.7 and 11.4 Hz, 1 H, 5-H^B, **B**), 3.76 (dd, J = 13.2 and 10.5 Hz, 1 H, 5-H^B, **A**), 4.13 (dd, J = 10.5 and 8.4 Hz, 1 H, NCH, **A**), 4.19/4.33 (AB quartet, ^2J = 13.1 Hz, 2 H, SCH_2Ph, **A**), 4.23–4.31 (m, 2 H, OCH_2), 4.64/4.95 (AB quartet, ^2J = 12.2 Hz, 2 H, SCH_2Ph, **B**), 5.15 (dd, J = 11.1 and 6.4 Hz, 1 H, NCH, **B**), 7.33–7.53 (m, 5 H, H_{Ph}), 7.65–7.69 (m, 2 H, H_{Pht}), 7.74–7.76 (m, 2 H, H_{Pht}), 7.86–7.88 (m, 2 H, H_{Pht}) ppm. MS (CI): m/z (%) = 410 (33, $[M]^+$ for $C_{22}H_{19}O_5S$). MS (HRMS-ESI): m/z found: 432.0885; calcd. for $C_{22}H_{19}NO_5S$ + Na: 432.0882.

Dimer 13b: White solid, mp 206–212 °C. IR (KBr): 3059 w, 3027 w, 2979 w, 1755 s, 1722 s, 1360 s cm^{-1}. ^{1}H NMR ($CDCl_3$): δ = 0.70 (t, J = 7.1 Hz, 3 H, OCH_2CH_3), 2.85 (A part of ABX system, ^2J = 14.0 Hz, ^3J = 9.8 Hz, 1 H, NCHC*H*H), 3.33–3.41 and 3.60–3.68 (2 m, 1 H each, OCH_2), 3.56 (B part of ABX system, ^2J = 14.0 Hz. ^3J = 4.9 Hz, 1 H, NCHCH*H*), 3.65 (s, 2 H, $PhCH_2S$), 4.67 (X part of ABX system, ^3J = 9.8 and 4.9 Hz, 1 H, NCH), 7.21–7.33 (m, 5 H, H_{Ph}), 7.61–7.68 (m, 2 H), 7.72–7.77 (m, 1 H, H_{Pht}), 7.87–7.91 (m, 1H, H_{Pht}) ppm. ^{13}C NMR ($CDCl_3$): δ = 13.38, 30.60, 36.19, 63.45, 64.58, 86.81, 94.09, 122.89, 123.93, 127.46, 128.73, 129.11, 131.65, 132.65, 133.08, 137.16, 141.99, 161.07, 166.60, 193.97 ppm. MS (CI): m/z (%) = 819 (13, $[M]^+$), 410 (100). Anal. calcd. for $C_{44}H_{38}N_2O_{10}S_2$ (818.92): C, 64.53; H, 4.68; N, 3.42; found: C, 64.13; H, 4.76; N, 3.46.

Decomposition of 5-allylsulfanyl-2-diazo-3-oxo-4-phthalimidopentanoic acid ethyl ester ((±)-8c)

Following the procedure described for the decomposition of **8a**, the crude reaction product was a red oil, which was subjected to column chromatography (eluent: cyclohexane–ethyl acetate (2:1)). Two major products, **16** and **17**, were isolated.

2-Allyl-3-oxo-4-phthalimido-tetrahydrothiophene-2-carboxylic acid ethyl ester (16): Yellow oil, mixture of diasteromers (3:2 by ^{1}H NMR integration); yield: 31%. IR (film): ν = 2982 w, 2907 w, 1754 s, 1721 s, 1387 s cm^{-1}. ^{1}H NMR ($CDCl_3$), isomer **A** (major) and **B** (minor): δ = 1.31 (t, *J* = 7.1 Hz, 3 H, OCH_2CH_3, **B**), 1.38 (t, *J* = 7.1 Hz, 3 H, OCH_2CH_3, **A**), 2.67–2.73 (m, =CH–C*H*H of **A**), 2.90–3.04 (m, =CH–CH*H* of **A**, =CH–C*HH* of **B**)), 3.08–3.16 (m, 1 H, SC*H*H, **A** and **B**), 3.45–3.50 (virtual t, 1 H, SCH*H*, **B**), 3.73–3.78 (virtual t, 1 H, SCH*H*, **A**), 4.20–4.28 (m, 2 H, OCH_2, **B**), 4.34 (q, *J* = 7.1 Hz, 2 H, OCH_2, **A**), 5.00 (dd, ^{3}J = 11.7 and 8.0 Hz, 1 H, NCH, **A**), 5.16–5.21 (m, 2 H, CH_2=CH, **A**), 5.24–5.29 (m, 2 H, CH_2=CH, **B**), 5.40 (dd, ^{3}J = 11.0 and 9.0 Hz, 1 H, NCH, **B**), 5.77–5.87 (m, 1 H, CH_2=C*H*, **A**), 5.90–6.00 (m, 1 H, CH_2=C*H*, **B**), 7.65–7.72 (m, 2 H, H_{Pht}, **A** and **B**), 7.76–7.83 (m, 2 H, H_{Pht}, **A** and **B**) ppm. ^{13}C NMR ($CDCl_3$), isomer **A**/isomer **B**: δ = 14.13/14.05, 27.38/24.49, 39.76/39.58, 57.09/56.65, 61.94/59.66, 62.95/62.77, 120.59/120.49, 123.84/123.87, 131.61/131.64, 131.87/131.91, 134.54/134.61, 166.90/167.08, 168.75/169.14, 202.05/200.82 ppm. MS (CI): *m*/*z* (%) = 388 (23), 360 (100, [M]$^{+}$), 286 (31). Anal. calcd. for $C_{18}H_{17}NO_5S$: C, 60.15; H, 4.77; N, 3.90; found: C, 60.07; H, 4.81; N, 3.70.

Pentacycle 17: Yellow oil; yield: 35%. IR (film): ν = 2988 w, 2938 w, 2917 w, 1745 s, 1715 s, 1383 s cm^{-1}. ^{1}H NMR ($CDCl_3$): δ = 1.34 (t, *J* = 7.1 Hz, 3 H, OCH_2CH_3), 2.68 (dd, *J* = 12.7 and 11.5 Hz, 1 H, OCC*H*H), 2.90–2.96 (m, 3 H, NCHC*H*HS, CH-bridgehead), 3.09 (dd, *J* = 13.5 and 1.6 Hz, Hz, 1 H, NCHCH*H*S), 3.14 (dd, *J* = 12.7 and 2.0 Hz, OCCH*H*), 3.58–3.64 (m, 1 H, SCH_2C*H*), 4.28–4.44 (m, 2 H, OCH_2), 5.20 (dd, ^{3}J = 6.1 and 1.7 Hz, 1 H, NCH), 7.57–7.62 (m, 1 H, H_{Pht}), 7.63–7.68 (m, 2 H, H_{Pht}), 7.84–7.86 (m, 1 H, H_{Pht}) ppm. ^{13}C NMR ($CDCl_3$): δ = 14.27, 32.31, 33.95, 37.16, 38.25, 62.85, 63.52, 92.38, 96.47, 123.09, 124.19, 131.13, 132.97, 132.97, 140.58, 166.25, 167.73, 193.97 ppm. MS (CI): *m*/*z* (%) = 388 (16), 360 (100, [M]$^{+}$). Anal. calcd. for $C_{18}H_{17}NO_5S$ (359.40): C, 60.16; H, 4.77; N, 3.90; found: C, 59.80; H, 4.84; N, 3.89.

Rhodium-catalysed decomposition of diazoester 8a in the presence of *N*-phenylmaleimide

To a suspension of $Rh_2(OAc)_4$ (13 mg, 0.03 mmol) and *N*-phenylmaleimide (0.173 g, 1.00 mmol) in dry benzene (20 mL) under reflux was added a solution of racemic diazoester **8a** (0.315 g, 1.00 mmol) in dry benzene (5 mL) through a syringe pump over a period of 1 h. When the diazo compound had been consumed (2 h, IR control, disappearance of the ν(CN_2) absorption), the solvent was evaporated (last traces at 25 °C/10^{-3} mbar). The resulting red

solid was triturated with cyclohexane–ethyl acetate (1:1). The insoluble residue, consisting of cycloadduct **18**, was filtered off and dried at 25 °C/10^{-3} mbar to leave a white solid (0.20 g, 0.38 mmol, 38% yield), mp 168–172 °C (dec.). IR (KBr): ν = 3067 w, 2982 w, 2907 w, 1717 s, 1386 m cm^{-1}. ^{1}H NMR ($CDCl_3$): δ = 1.37 (t, *J* = 7.1 Hz, 3 H, OCH_2CH_3), 1.80 (broadened s, 3 H, SCH_3), 2.34–2.41 (m, 1 H, NCHC*H*H), 2.52–2.62 (m, 2 H, SCH_2), 3.26–3.33 (m, 1 H, NCHCH*H*), 3.82 (d, *J* = 7.4 Hz, 1 H, CH bridgehead), 4.04 (d, *J* = 7.4 Hz, 1 H, CH bridgehead), 4.36–4.44 (m, 2 H, OCH_2), 4.60–4.61 (m, 1 H, NCH), 7.27–7.28 (m, 1 H, H_{Pht}), 7.33–7.35 (m, 2 H, H_{Pht}), 7.43–7.62 (m, 5 H, H_{Ph}), 7.83–7.85 (m, 1 H, H_{Pht}) ppm. ^{13}C NMR ($CDCl_3$): δ = 14.24, 14.41, 28.73, 29.40, 52.03, 54.41, 60.08, 62.93, 90.06, 95.86, 123.97, 124.24, 126.19, 129.47, 129.68, 131.20, 131.52, 132.38, 132.48, 137.44, 162.24, 165.07, 171.00, 171.06, 191.96 ppm. MS (HRMS-ESI, complexation with $AgNO_3$ [11]): *m*/*z* found: 627.0291; calcd. for $C_{27}H_{24}N_2O_7S$ + Ag: 627.0355.

Rhodium-catalysed decomposition of diazoester 8b in the presence of DMAD

To a suspension of $Rh_2(OAc)_4$ (13 mg, 0.03 mmol) and dimethyl acetylenedicarboxylate (0.12 mL, 1.00 mmol) in dry benzene (20 mL) under reflux was added a solution of racemic diazoester **8a** (0.375 g, 1.00 mmol) in dry benzene (5 mL) through a syringe pump over a period of 1 h. After 2 h the diazo compound had been consumed (IR control, disappearance of the ν(CN_2) absorption). Evaporation of the solvent (last traces at 25 °C/10^{-3} mbar) left a highly viscous red oil consisting of cycloadduct **19** (2:1 mixture of two diastereomers), residual DMAD, and the catalyst. Efforts towards further purification were not successful. Spectroscopic data of **19**: IR (film): ν = 2983 w, 2955 w, 2929 w, 1720 s, 1377 s cm^{-1}. ^{1}H NMR ($CDCl_3$), isomer **A** (major) and **B** (minor): δ = 1.33 (t, *J* = 7.2 Hz, 3 H, OCH_2CH_3, **A** and **B**), 1.98 (broadened s, 3 H, SCH_3, **A**), 2.13 (broadened s, 3 H, SCH_3, **B**), 2.33–2.48 (m, 1 H)/2.53–2.75 (m, 2 H)/3.24–3.31 (m, 1 H) (SCH_2CH_2, **A** and **B**), 3.52 (s, 3 H, OCH_3, **A**), 3.63 (s, 3 H, OCH_3, **B**), 3.92 (s, 3 H, OCH_3, **A**), 3.96 (s, 3 H, OCH_3, **B**), 4.29–4.44 (m, 2 H, OCH_2, **A** and **B**), 4.87 (dd, *J* = 5.3 and 2.5 Hz, 1 H, NCH, **A**), 5.03 (dd, *J* = 8.1 and 6.1 Hz, 1 H, NCH, **B**), 7.61–7.68 (m, 3 H, H_{Pht}), 7.83–7.87 (m, 1 H, H_{Pht}) ppm. ^{13}C NMR ($CDCl_3$), isomer **A**: δ = 14.07, 15.46, 30.84, 36.68, 53.19, 53.53, 61.49, 63.51, 92.92, 97.92, 124.19, 124.34, 131.67, 132.19, 133.13, 135.89, 139.25, 144.04, 159.77, 161.90, 162.34, 167.09, 189.57 ppm. MS (CI): *m*/*z* (%) = 518 (27), 490 (100, $[M]^+$ for $C_{23}H_{23}NO_9S$), 414 (13).

Catalytic decomposition of α-diazo-β-ketoesters 11a,b

Decomposition of 2-diazo-6-methylsulfanyl-3-oxohexanoic acid ethyl ester (11a)

To a suspension of $Rh_2(OAc)_4$ (13 mg, 0.03 mmol) in dry benzene (20 mL) under reflux was added a solution of diazoester **11a** (0.230 g, 1.00 mmol) in dry benzene (5 mL) through a syringe pump over a period of 1 h. After 4 h the diazo compound had been consumed (IR control, disappearance of the ν(CN_2) absorption). Evaporation of the solvent (last traces at 25 °C/10^{-3} mbar) left a greenish solid, which was triturated with ethyl acetate. The insoluble residue, *1-methyl-3-oxo-3,4,5,6-tetrahydro-1λ⁴-thiapyran-2-carboxylic acid ethyl ester* (**20**), was filtered off and dried at 25 °C/10^{-3} mbar; white solid, mp 192–197 °C (dec.); yield: 0.198 g (0.98 mmol, 98%). IR (KBr): ν = 2977 s, 2949 s, 2900 s, 1617 s, 1555 s, 1380 s cm^{-1}. ^1H NMR (CD_3CN): δ = 1.21 (t, J = 7.1 Hz, 3 H, OCH_2CH_3), 2.10–2.16 (m, 1 H, SC*H*H), 2.19–2.23 (m, 2 H, SCH_2CH_2), 2.28–2.39 (m, 1 H, SCH*H*), 2.62 (s, 3 H, SCH_3), 3.05–3.11 (m, 1 H, C*H*HCO), 3.21–3.28 (m, 1 H, CH*H*CO), 4.02–4.16 (m, 2 H, OCH_2) ppm. ^{13}C NMR (CD_3CN): δ = 15.11, 19.69, 30.23, 35.65, 37.48, 59.65, 73.52, 167.24, 182.99 ppm. MS (CI): *m*/*z* (%) = 231 (23), 203 (69, $[M]^+$), 187 (4), 157 (100). Anal. calcd. for $C_9H_{14}O_3S$ (202.27): C, 53.44; H, 6.98; found: C, 53.62; H, 6.91.

Decomposition of 5-benzylsulfanyl-2-diazo-3-oxopentanoic acid ethyl ester (11b)

To a suspension of $Rh_2(OAc)_4$ (13 mg, 0.03 mmol) in dry benzene (20 mL) under reflux was added a solution of diazo ester **11b** (0.292 g, 1.00 mmol) in dry benzene (5 mL) through a dropping funnel over a period of 5 min. After 30 min the diazo compound had been consumed (IR control, disappearance of the ν(CN_2) absorption), and the solvent was evaporated (last traces at 25 °C/10^{-3} mbar). The resulting redish solid was triturated with ethyl acetate. The insoluble residue, sulfur ylide **21,** was filtered off and dried at 25 °C/10^{-3} mbar. The filtrated liquid was evaporated to leave an oil, which was subjected to column chromatography (cyclohexane–ethyl acetate (8:1)) to obtain dienol **22**.

1-Benzyl-3-oxo-4,5-dihydro-3*H*-1λ⁴-thiophene-2-carboxylic acid ethyl ester (21): off-white solid, mp 160–165 °C; yield: 0.207 g (0.78 mmol, 78%). IR (KBr): 3064, 3020, 2987, 2958, 2931 (all w), 1687 s, 1373 m cm^{-1}. ^1H NMR ($CDCl_3$): δ = 1.35 (t, J = 7.1 Hz, 3 H, OCH_2CH_3), 2.04–2.13/2.36–2.42/2.94–3.00/3.16–3.24 (4 m, 1 H each, CH_2CH_2), 4.04 and 4.18 (AB quartet, ^2J = 13.0 Hz, 2 H, $PhCH_2$), 4.22–4.38 (m, 2 H, OCH_2), 7.27–7.29 (m, 2 H, H_{Ph}), 7.40–7.46 (m, 3 H, H_{Ph}) ppm. ^{13}C NMR ($CDCl_3$): δ = 14.96, 28.90, 34.45, 50.03, 60.23, 73.35, 127.96, 129.69, 129.91, 130.42, 164.34, 187.34 ppm. MS (CI): *m*/*z* (%) = 293 (17), 265

(100, [M]$^+$), 219 (55), 191 (58), 173 (16), 91 (41). Anal. calcd. for $C_{14}H_{16}O_3S$ (264.34): C, 63.61; H, 6.10; found: C, 63.78; H, 6.12.

2-Benzylsulfanyl-3-hydroxypenta-2,4-dienoic acid ethyl ester (22): Obtained as a red oil, which contained some impurities (^{1}H NMR); yield: 4%: ^{1}H NMR (400.13 MHz): δ = 1.35 (t, *J* = 7.1 Hz, 3 H, OCH_2CH_3), 3.72 (s, 2 H, $PhCH_2$), 4.26 (q, *J* = 7.1 Hz, 2 H, OCH_2), 5.47 (dd, *J* = 10.8 and 1.6 Hz, 1 H, C*H*H=CH), 6.07 (dd, *J* = 17.2 and 1.6 Hz, 1 H, CH*H*=CH), 6.99 (ddd, J = 17.2, 10.8 and 1.6 Hz, 1 H, CH_2=C*H*), 7.12–7.14 (m, 2 H, H_{Ph}), 7.22–7.25 (m, 3 H, H_{Ph}), 13.29 (d, *J* = 1.6 Hz, 1 H, OH-enol) ppm. ^{13}C NMR ($CDCl_3$): δ = 14.21, 40.50, 61.67, 124.54, 126.91, 127.96, 128.34, 129.07, 131.16, 137.52, 173.38, 174.51 ppm. MS (CI): *m*/*z* (%) = 265 (69, [M]$^+$ for $C_{14}H_{16}O_3S$), 219 (51), 191 (42), 173 (23), 91 (64).

References

1. Koerber-Ple, K.; Massiot, G. *J. Heterocycl. Chem.* **1995**, *32*, 1309–1316.
2. Freeman, F.; Huang, B.-G. *J. Org. Chem.* **1994**, *59*, 3227–3229.
3. Williams, K. A.; Doi, J. T. ; Musker, W. K. *J. Org. Chem.* **1985**, *40*, 4–10.
4. Angelosante, J. K.; Lewis, B. J.; Cooper, L. E.; Swanson, R. A.; Daley, C. J. A. *Phosphorus Sulfur Silicon Relat. Elem.* **2006**, *181*, 2263–2272.
5. Goddard-Borger, E. D.; Stick, R. V. *Org. Lett.* **2007**, *9*, 3797–3800.
6. Bose, A. K.; Greer, F.; Price, C. C. *J. Org. Chem.* **1958**, *23*, 1335–1338.
7. Bose, A. K. *Org. Synth. Coll. Vol. 5* **1973**, 973–975.
8. Guttmann, S.; Boissannas, R. A. *Helv. Chim. Acta* **1958**, *41*, 1852–1867.
9. Griesbeck, A. G.; Hirt, J. *Liebigs Ann. Chem.* **1995**, *11*, 1957–1962.
10. King, F. E.; Clark-Lewis, J. W.; Wade, R. *J. Chem. Soc.* **1957**, 886–894.
11. Bayer, E.; Gfrörer, P.; Rentel, C. *Angew. Chem.* **1999**, *111*, 1046–1049; *Angew. Chem. Int. Ed.* **1999**, *38*, 992–995.
